# Kushenin Combined with Nucleos(t)ide Analogues for Chronic Hepatitis B: A Systematic Review and Meta-Analysis

**DOI:** 10.1155/2015/529636

**Published:** 2015-08-11

**Authors:** Zhe Chen, Xiao Ma, Yanling Zhao, Jiabo Wang, Yaming Zhang, Yun Zhu, Lifu Wang, Chang Chen, Shizhang Wei, Zhirui Yang, Man Gong, Honghui Shen, Zhaofang Bai, Yuming Guo, Ming Niu, Xiaohe Xiao

**Affiliations:** ^1^China Military Institute of Chinese Medicine, 302 Hospital of People's Liberation Army, Beijing 100039, China; ^2^Pharmacy College, Chengdu University of Traditional Chinese Medicine, Chengdu 611137, China; ^3^Department of Integrative Medical Center, 302 Hospital of People's Liberation Army, Beijing 100039, China

## Abstract

*Objective*. To evaluate the efficacy and safety of Kushenin (KS) combined with nucleoside analogues (NAs) for chronic hepatitis B (CHB). *Methods*. Randomized controlled trials (RCTs) of KS combined with NAs for CHB were identified through 7 databases. Frequencies of loss of serum HBeAg, HBeAg seroconversion, undetectable serum HBV-DNA, ALT normalization, and adverse events at 48 weeks were abstracted by two reviewers. The Cochrane software was performed to assess the risk of bias in the included trials. Data were analyzed with Review Manager 5.3 software. *Results*. 18 RCTs involving 1684 subjects with CHB were included in the analysis. KS combined with NAs including lamivudine (LAM), entecavir (ETV), adefovir dipivoxil (ADV), and telbivudine (TLV) showed different degree of improvement in CHB indices. KS combined with NAs increased the frequency of loss of serum HBeAg, HBeAg seroconversion, undetectable HBV-DNA levels, and ALT normalization compared with single agents. It also decreased serum ALT and AST level after one-year treatment. However, KS combined with TLV did not show a significant difference in CHB indices. The side-effects of KS combined with NAs were light and of low frequency. *Conclusion*. KS combined with NAs improves the efficacy of NAs in CHB.

## 1. Introduction

Chronic hepatitis B (CHB) is a global health problem with an estimated 400 billion people worldwide being chronically infected with the virus (HBV) [1]. Chronic infection with HBV can significantly impair the quality of life and life expectancy of patients. Disease progression can lead to fibrosis, cirrhosis, liver failure, and hepatocellular carcinoma [2]. Eventually, approximately 25% of the infected patients will die of the liver-related complications if untreated [3]. There is a high frequency of chronic hepatitis B virus infection in China. Approximately 60% of the population have a history of HBV infection. An estimated 7%–10% of people are chronically infected with HBV and are at increased risk of premature death from liver diseases [4].

Currently, available treatments include interferon-alfa, nucleos(t)ide analogue polymerase inhibitors, such as lamivudine (LAM), adefovir (ADV), entecavir (ETV), telbivudine, and tenofovir (TLV), used alone or in combination with 2 or more agents [3]. Interferon is used as a short-term treatment that, if successful, may lead to long-term immune control without the need for further antiviral therapy [5]. Nucleos(t)ide analogues (NAs) directly inhibit HBV reverse-transcriptase polymerase. However, with such antiviral therapy HBV replication increases markedly as soon as the treatment is stopped [6]. Furthermore, with long-term use viral resistance often emerges and may eventually create serious clinical problems [7]. Therefore, strategies for enhancing the efficacy and safety of these agents are key aspects of CHB treatment.

Traditional Chinese medicine (TCM), one of the complementary and alternative therapies, is often used in cholestasis treatment in China [8]. The* Yellow Emperor's Internal Classic*, an ancient book containing records of TCM, indicates that TCM was used to treat chronic liver disease in China since 475 BCE at least [9]. The objectives of TCM treatment of CHB are to (i) relieve anxiety and symptoms and hence improve the quality of life of the patients, (ii) alleviate inflammation, (iii) arrest hepatic fibrosis, (iv) improve immune function, and (v) improve lipid metabolism [9].

Kushenin (KS) is a mixture of the alkaloids oxymatrine (the main content > 98%) and oxysophocarpine, extracted from the root of* Sophora flavescens* Alt. (*Kushen* in Chinese). Evidence-backed studies reveal its effectiveness in treating hepatocyte injury, chronic hepatitis B, liver fibrosis, and tissue inflammation [10]. It has been reported that a single-therapy treatment may result in the emergence of both viral drug-resistance and dose-dependent side- effects [11]. Under this circumstance, some physicians have attempted to use combinations of TCM with LAM to enhance its curative effect. Recently, we note that the clinical combination of KS with LAM could improve curative effect with regard to HBV infection [12–17]. It has been reported that the combination of KS with LAM could significantly increase the negative conversion rate of HBV-DNA and hepatitis B e antigen (HBeAg) in patients compared with LAM treatment. It has also been reported that KS could decrease the development of drug resistance to LAM [10].

Nowadays, the combinations of KS with NAs for CHB treatment have attracted more and more attention. It demonstrates a promising therapy of the combinations of KS with NAs for CHB treatment in clinic. Therefore, this meta-analysis of RCTs was conducted to assess the clinical value of KS combined with NAs for the treatment of CHB, which provides a possible complementary and alternative therapy for global application.

## 2. Materials and Methods

### 2.1. Search Strategy

Comprehensive searches of English and Chinese databases were performed by two researchers. The databases included PubMed, Embase, Cochrane Library, Chinese Biomedical Database (CBM), Wanfang, VIP medicine information system (VMIS), and China National Knowledge Infrastructure (CNKI), and the dates ranged from the establishment of the different databases through 2015. Search terms included Kushenin, chronic hepatitis B, HBV, CHB, and randomized controlled trial. The first author, year of publication, title, and journal name of the articles were recorded for further screening.

### 2.2. Inclusion Criteria

The inclusion criteria were as follows. (1) There are randomized controlled trials (RCTs). (2) The diagnostic criteria for CHB were serum hepatitis B surface antigen (HBsAg) and hepatitis B e antigen (HBeAg) positive for more than 6 months, with elevating levels of serum alanine aminotransferase (ALT). (3) Studies were selected for analysis if there was an objective outcome, including ALT normalization, loss of serum HBeAg, and/or undetectable serum HBV-DNA. (4) Intervention therapies with combinations of KS with NAs or single application were included.

### 2.3. Exclusion Criteria

The exclusion criteria were as follows. (1) There are studies of patients who were coinfected with HIV, HCV, or HDV. (2) Patients did not present any severe complications, such as hepatic failure and cirrhosis. (3) Studies did not report any efficacy measures or not conveying sufficient statistical information. (4) There are studies without equal baseline or endpoint of outcome measure.

### 2.4. Data Extraction and Risk of Bias Assessment

Data extraction and quality assessment were independently performed by two researchers and disagreements were resolved by consensus. Detailed data such as data source, eligibility, methods, participants, interventions, and study results were imported into Cochrane Review Manager 5.3 (The Nordic Cochrane Centre, The Cochrane Collaboration, Copenhagen, 2014) for further analysis.

The Cochrane risk of bias tool was used to assess the methodological quality of included RCTs. The six domains of this tool include random sequence generation (selection bias), allocation concealment (selection bias), blinding of participants and personnel (performance bias), blinding of outcome data (attrition bias), incomplete outcome data (attrition bias), and selective reporting (reporting bias). The judgment was marked as “high risk,” “unclear risk,” or “low risk.” Trials that met all the criteria were categorized as low risk of bias, whereas those that met none were categorized as high risk of bias. The others were classified as unclear risk of bias if the information was insufficient to make a judgment.

### 2.5. Data Analysis

Statistical analysis was performed by Cochrane Review Manager 5.3 (The Nordic Cochrane Centre, The Cochrane Collaboration, Copenhagen, 2014). Dichotomous data were presented as odds ratio (OR) and continuous variables as mean difference (MD), with 95% confidence intervals (95% CI). Statistical heterogeneity was assessed by Cochrane's *Q* test. Only data with low heterogeneity (*P* ≥ 0.10 and *I* ^2^ ≤ 50%) were assessed as a fixed-effects model whereas others were assessed as a random-effects model. A funnel plot was used for assessing the potential publication bias.

## 3. Results

### 3.1. Characteristics of Included Trials

**Figure 1:**
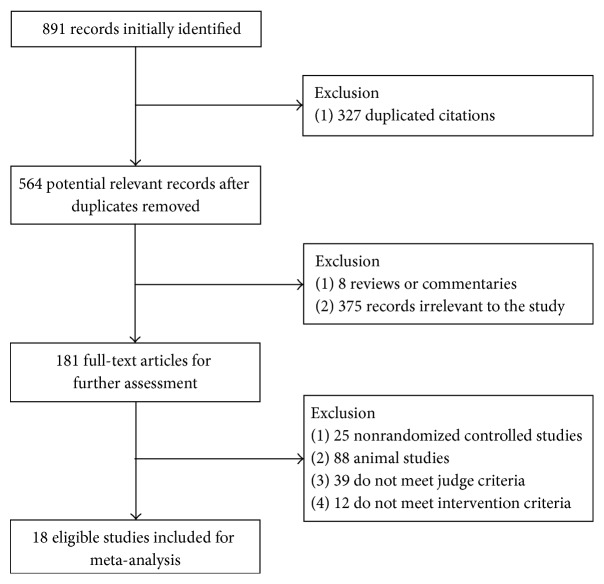
Flowchart of study selection.

A total of 891 records were identified for initial screening and 18 eligible articles were included in this meta-analysis (Figure 1). There was no significant difference in ages, sex, and course of disease between two groups. Of these 18 articles, 6 were treated with combination of KS and LAM, 4 were treated with combination of KS and ETV, 5 were treated with combination of KS and ADV, and 3 were treated with combination of KS and TLV. Seven of 18 studies were reported with slight adverse events (Table 1).

### 3.2. Methodological Quality of Included Trials

**Figure 2:**
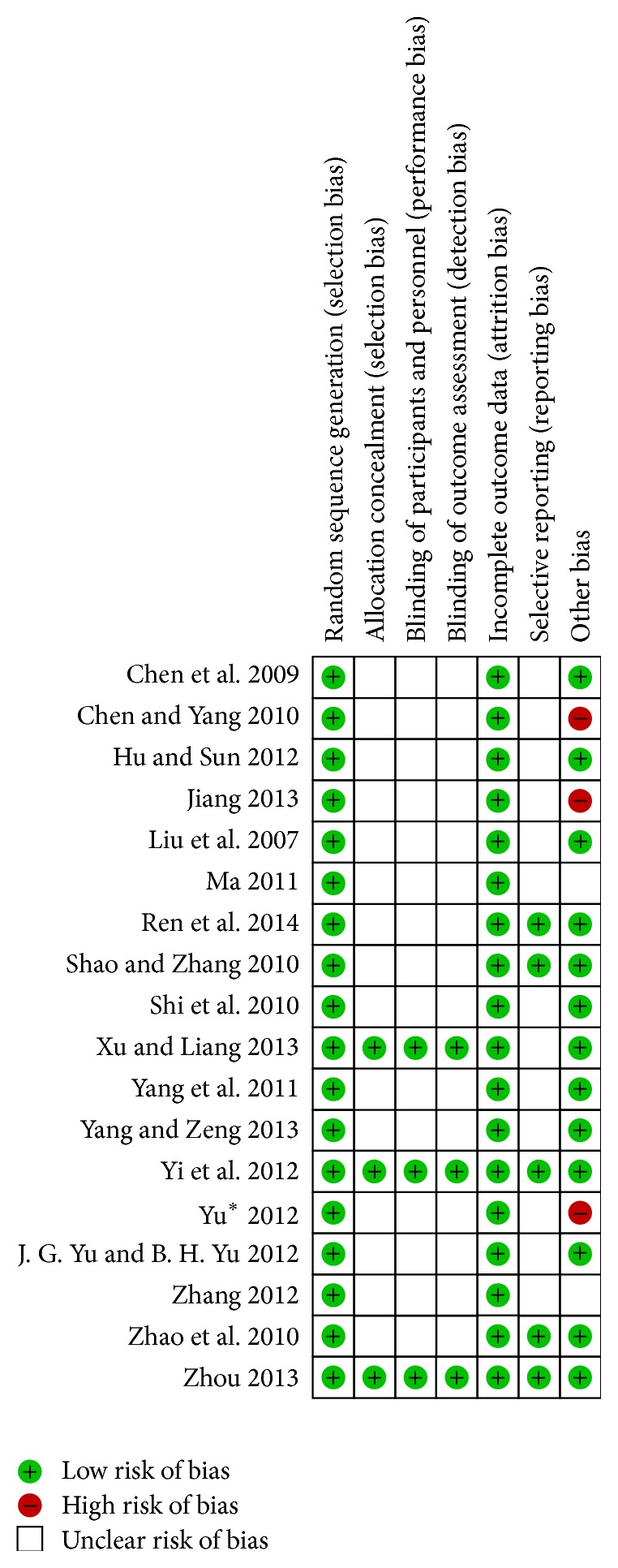
Methodological quality assessment of the risk of bias for each included study.

According to Cochrane risk of bias estimation, randomized allocation of participants was mentioned in all trials. All the trials were classified using a random number table [12–29]. Three of 18 trials performed allocation concealment, blinding of participants, and personnel assessment and blinding of outcome assessment [17, 19, 26]. There was no incomplete outcome data or essential data missing [12–29]. Five articles reported systematic project in research [12, 17–19, 21] and others remained unclear (Figure 2).

### 3.3. Outcome Measures

#### 3.3.1. Loss of Serum HBeAg Rate

**Figure 3:**
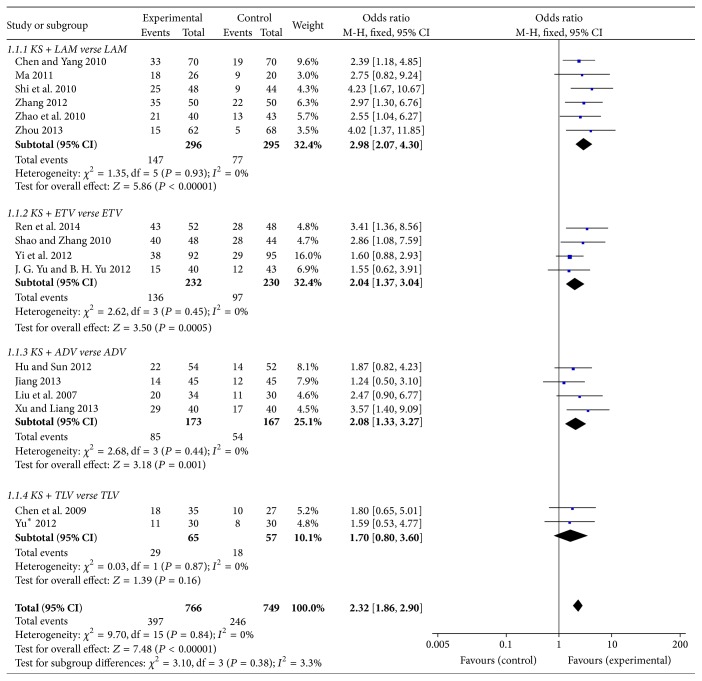
Loss of serum HBeAg rate of KS combined with NAs.

Loss of serum HBeAg rate is a crucial terminal index of anti-HBV therapy. After 48-week treatment, KS + LAM group showed a significant effect on loss of serum HBeAg rate compared with LAM group (OR = 2.98, 95%  CI = 2.07–4.30; *P* < 0.00001). KS + ETV and KS + ADV group also suggested a significant effect on it and their values were OR = 2.04, 95%  CI = 1.37–3.04, *P* = 0.0005 and OR = 2.08, 95%  CI = 1.33–3.27, *P* = 0.001, respectively. However, there was no significance in KS + TLV group, which could be caused by the small scale of the patients (OR = 1.70, 95%  CI = 0.8–3.6; *P* = 0.16) (Figure 3).

#### 3.3.2. HBeAg Seroconversion Rate

**Figure 4:**
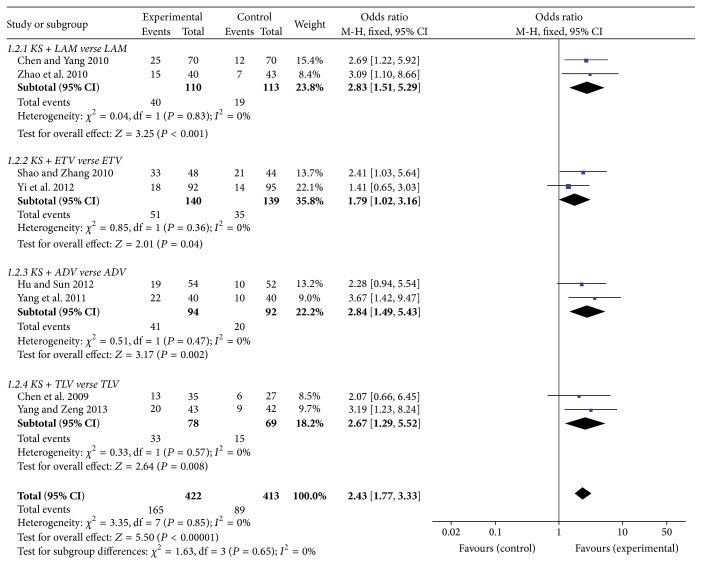
HBeAg seroconversion rate of KS combined with NAs.

HBeAg seroconversion may lead to HBsAg seroclearance, which is the most desirable endpoint and reflects “cure” of chronic hepatitis B [30]. The HBeAg seroconversion rate of KS + LAM group indicated a higher rate than single LAM (OR = 2.83, 95%  CI = 1.51–5.29; *P* = 0.001). KS + ETV group showed a significant effect on HBeAg seroconversion rate compared with ETV group (OR = 1.79, 95%  CI = 1.02–3.16; *P* = 0.04). KS + ADV group also exhibited a significant effect on it and the values were OR = 2.84, 95%  CI = 1.49–5.43, and *P* = 0.002. However, there was no significance in KS + TLV group, which could be caused by small scale of the patients (OR = 2.67, 95%  CI = 1.29–5.52; *P* = 0.08) (Figure 4).

#### 3.3.3. Undetectable Serum HBV-DNA Rate

**Figure 5:**
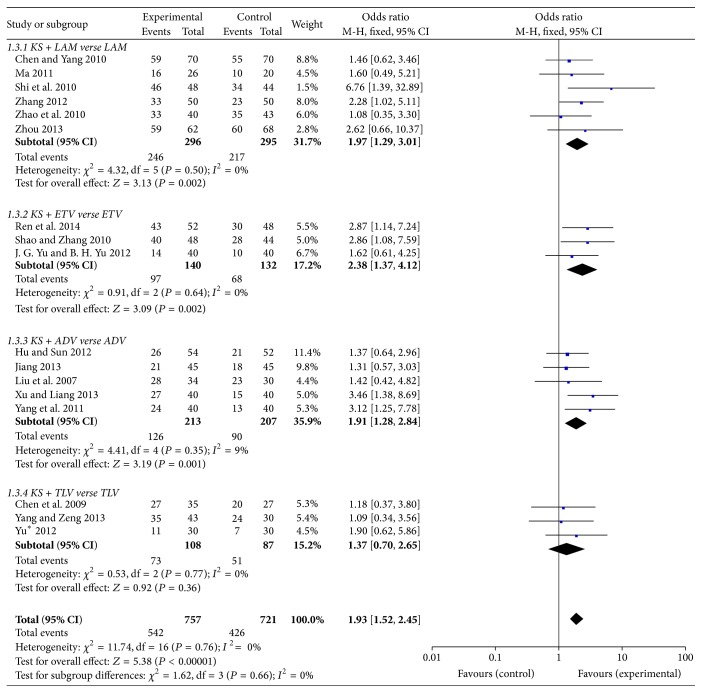
Undetectable serum HBV-DNA rate of KS combined with NAs.

In order to explore the effect on undetectable serum HBV-DNA rate, we studied the combination of KS with four frequently used anti-HBV agents (LAM, ETV, ADV, and TLV). After 48-week treatment, 83.1% of patients in KS + LAM group and 73.5% of patients in LAM group reached undetectable serum HBV-DNA levels, and there was a significant difference between two groups (OR = 1.97, 95%  CI = 1.29–3.01; *P* = 0.002). 69.3% of patients in KS + ETV group and 51.5% of patients in ETV group reached undetectable serum HBV-DNA levels, and there was also a significant difference between two groups (OR = 2.38, 95%  CI = 1.37–4.12; *P* = 0.002). Moreover, 54.5% of patients in KS + ADV group and 43.5% of patients in ADV group reached the undetectable levels, and KS + ADV group showed a significant effect on undetectable serum HBV-DNA rate at 48 weeks (OR = 1.91, 95%  CI = 1.28–2.84; *P* = 0.001), whereas there was no significance in KS + TLV and TLV group (OR = 1.37, 95%  CI = 0.70–2.65; *P* = 0.36), which could be caused by small scale of the patients (Figure 5).

#### 3.3.4. ALT Normalization Rate

**Figure 6:**
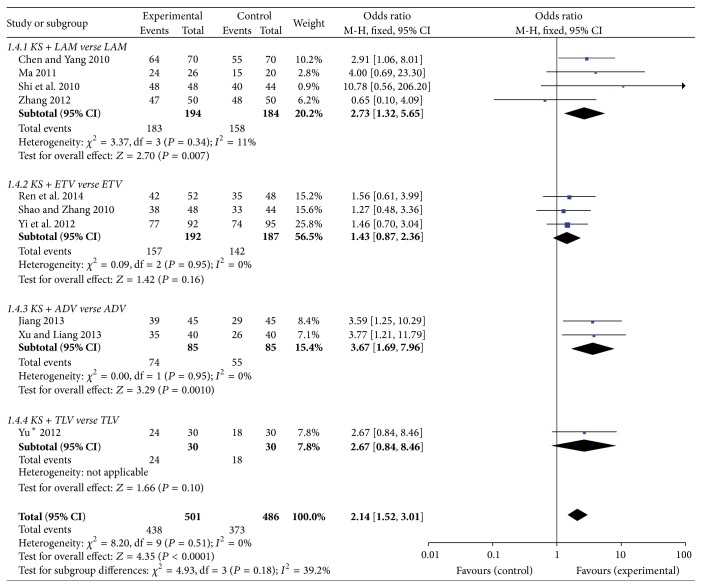
ALT normalization rate of KS combined with NAs.

To explore the effect on undetectable serum HBV-DNA rate, we studied the combination of KS with four frequently used anti-HBV agents (LAM, ETV, ADV, and TLV). After 48-week treatment, 94.3% of patients in KS + LAM group and 85.9% of patients in LAM group got ALT normalization. There was a significant difference between those two groups (OR = 2.73, 95%  CI = 1.32–5.65; *P* = 0.007). ALT normalization rate of KS + ADV reached 87.1% and ADV alone reached 64.7% at 48 weeks, indicating a significant difference between two groups (OR = 3.67, 95%  CI = 1.69–7.96; *P* = 0.001). However, 81.8% of patients in KS + ETV group and 75.9% of patients in ETV group got ALT normalization. There was no significant difference between two groups (OR = 1.43, 95%  CI = 0.87–2.36; *P* = 0.16) (Figure 6).

#### 3.3.5. Serum ALT and AST Levels

**Figure 7:**
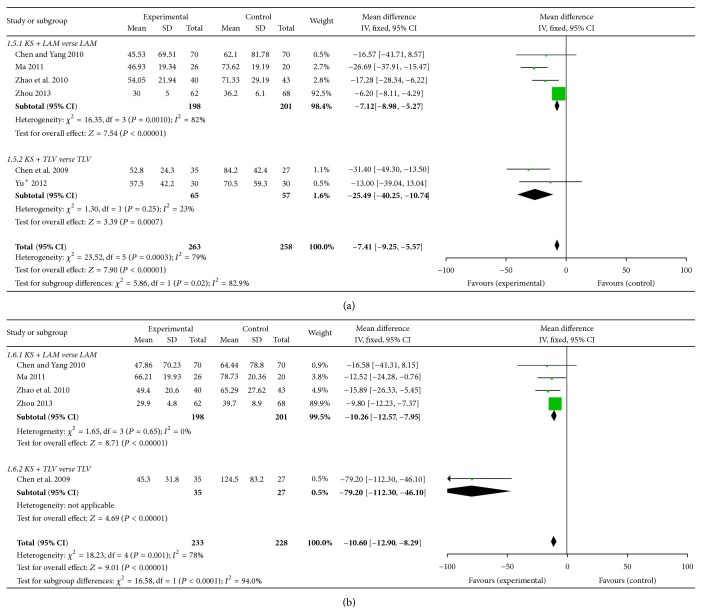
Serum ALT and AST level. (a) Serum ALT level of KS combined with NAs. (b) Serum AST level of KS combined with NAs.

Serum biochemical indices such as serum ALT, AST levels were detected. The results indicated that serum level of ALT and AST decreased lower in KS + LAM group than in LAM group at 48 weeks (MD = −7.12, 95%  CI = −8.89–−5.27; *P* < 0.00001 in ALT; MD = −10.26, 95%  CI = −12.57– − 7.95; *P* < 0.00001 in AST). Serum ALT and AST level also decreased lower in KS + TLV group than in TLV group at 48 weeks (MD = −25.49, 95%  CI = −40.25– − 10.74; *P* = 0.0007 in ALT; MD = −79.20, 95%  CI = −112.30– − 46.10; *P* < 0.00001 in AST). Both of KS + LAM and KS + TLV groups indicated a good favorable curative effect on decreasing serum ALT and AST levels (Figure 7).

### 3.4. Adverse Events

It was worth noting that no serious adverse event happened in the clinical trials. Among 18 RCTs, 5 trials displayed light digestive tract symptom and 2 trials showed dizziness or fatigue during treatment, but all the symptoms disappeared later. In spite of no difference between trial and control group, further investigation is needed for a systematic safety assessment of combinations of KS with NAs (Table 1).

## 4. Discussion

In this study, 18 RCTs involving 891 subjects with CHB were included. The combinations of KS with the main NAs therapy medicine including LAM, ETV, ADV, and TLV were conducted to explore the curative effect. Some CHB indices were selected to represent the curative effect. The loss of serum HBeAg and HBeAg seroconversion rate are considered as indicators of the patients transition to a state of substantially lower HBV replication, which when maintained is likely to be associated with improved long-term clinical outcomes and so are undetectable serum HBV-DNA and ALT normalization rate [31]. The indices of ALT, AST, and TBIL in the research focus more on the degree of liver damage.

In the meta-analysis, KS combined with LAM, ETV, or ADV showed a good curative effect after one-year treatment, which indicated that different improvement of chronic hepatitis B indices included loss of serum HBeAg, HBeAg seroconversion rate, and undetectable serum HBV-DNA. However, KS combined with TLV could enhance the loss of serum HBeAg, HBeAg seroconversion rate, undetectable serum HBV-DNA rate, and ALT normalization rate but did not show a significant difference. Serum ALT and AST level obviously decreased in combination therapy in meta-analysis, which suggested the hepatoprotective effects. This could be caused by small sample size and multicenter and large-scale randomized trials of KS combined with TLV needed are further carried out.

It is still difficult to draw firm conclusions since the studies reviewed here are flawed in some areas. First, the quality of the studies determines the quality of such analyses, and the studies included in this investigation had shortcomings in methodology. Second, the present meta-analysis was based on 18 published RCTs. The relatively small size of the study was a limitation since it restricts statistical power and may explain why some of the changes did not reach statistical significance. Furthermore, only one of the RCTs in this meta-analysis measured follow-up beyond 24 weeks and most of the RCTs did not measure follow-up. Due to this, statistical power of this analysis cannot define the long-term efficacy of combinations of KS with NAs.

Therefore, the methodological quality of clinical trials with KS combined with NAs for chronic hepatitis B needs to be improved. Rigorously designed, large randomized, double blind, placebo-controlled trials are required to confirm the efficacy of KS combined with NAs in chronic hepatitis B. The outcome measures should include logical changes, liver pathology, and endpoint events. Adverse events should be monitored by a standardized effective reporting system in clinical trials and rare serious adverse events can be observed through epidemiological studies.

## 5. Conclusions

The systematic review and meta-analysis indicate that the combinations of KS with NAs not only enhance the indexes of loss of serum HBeAg and HBeAg seroconversion, but also improve the undetectable serum HBV-DNA rate and ALT normalization rate to a certain degree. Meanwhile, there is no obvious adverse effect. In summary, KS combined with NAs could be a beneficial and safe treatment approach for patients to improve the comprehensive efficacy of CHB. Considering being accepted by more and more practitioners, further rigorously designed, multicenter, large-scale trials with higher quality worldwide are required.

## Figures and Tables

**Table 1:** The characteristics of included studies.

Author	Year	Cases T/C	Age (years) range, mean	Sex: male/female	Interventions		Duration (wk)/ follow-up (wk)	Adverse events	Outcome measures
					Trials group	Control group			
Zhao et al. [12]	2010	40/43	T: 16–62, 35 C: 16–62, 37	T: 25/15 C: 27/16	KS + LAM	LAM	48/NR	T: 4 cases and C: 5 cases with light digestive tract side-effect	HBeAg loss; HBeAg sero; HBV-DNA; ALT; AST; TER

Chen and Yang [13]	2010	70/70	T: 19–60, 34 C: 20–61, 33	T: 40/30 C: 42/28	KS + LAM	LAM	48/NR	NR	HBeAg loss; HBeAg sero; HBV-DNA; ALT norm; AST norm; ALT; AST

Shi et al. [14]	2010	48/44	T: 16–60, 32 C: 17–59, 32	T: 28/20 C: 25/19	KS + LAM	LAM	48/NR	Trials group had light digestive tract side-effect at first and then symptoms disappeared	HBeAg loss; HBeAg sero; HBV-DNA; ALT norm

Ma [15]	2011	26/20	T: 18–60 C: 18–60	30/16	KS + LAM	LAM	48/NR	0	HBeAg loss; HBV-DNA; ALT norm; AST norm; ALT; AST; TBIL

Zhang [16]	2012	50/50	T: 18–62, 36 C: 19–63, 36	T: 28/22 C: 29/21	KS + LAM	LAM	48/NR	0	HBeAg loss; HBV-DNA; ALT norm; AST norm

Zhou [17]	2013	62/68	T: 20–69, 42 C: 18–69, 40	T: 42/20 C: 46/22	KS + LAM	LAM	48/NR	NR	HBeAg loss; HBV-DNA; ALT; AST

Shao and Zhang [18]	2010	48/48	T: 19–64, 34 C: 19–64, 34	63/29	KS + ETV	ETV	48/NR	NR	HBeAg loss; HBeAg sero; HBV-DNA; ALT norm

Yi et al. [19]	2012	92/95	T: 18–65 C: 18–65	NR	KS + ETV	ETV	48/NR	T: 1 with light digestive tract side-effect	HBeAg loss; HBeAg sero; ALT norm

J. G. Yu and B. H. Yu [20]	2012	40/43	T: 18–50, 35 C: 18–50, 35	65/18	KS + ETV	ETV	48/NR	0	HBeAg loss; HBsAg loss; HBV-DNA; ALT norm

Ren et al. [21]	2014	52/48	T: 41–56, 47 C: 41–56, 47	53/47	KS + ETV	ETV	48/NR	T: 1 case and C: 2 cases with dizziness	HBeAg loss; HBV-DNA; ALT norm

Liu et al. [22]	2007	34/30	T: 16–65 C: 16–65	32/24	KS + ADV	ADV	48/24	0	HBeAg loss; HBsAg loss; HBV-DNA; TER

Yang et al. [23]	2011	40/40	T: 22–48, 37 C: 22–48, 37	48/32	KS + ADV	ADV	48/NR	Light digestive tract side-effect at first	HBeAg sero; HBV-DNA

Hu and Sun [24]	2012	54/52	T: 16–65, 29 C: 16–65	T: 38/16 C: 34/18	KS + ADV	ADV	48/NR	T: 4 cases and C: 2 cases with light digestive tract side-effect	HBeAg loss; HBeAg sero; HBV-DNA

Jiang [25]	2013	45/45	T: 19–49, 31 C: 18–52, 32	T: 30/15 C: 28/17	KS + ADV	ADV	48/NR	NR	HBeAg loss; HBV-DNA

Xu and Liang [26]	2013	40/40	NR	NR	KS + ADV	ADV	48/NR	T: 6 cases and C: 4 cases with dizziness and fatigue	HBeAg loss; HBV-DNA; ALT norm

Chen et al. [27]	2009	35/27	T: 29 C: 30	T: 24/11 C: 19/8	KS + TLV	TLV	48/NR	0	HBeAg loss; HBeAg sero; HBV-DNA; ALT; AST; TBIL

Yu^*∗*^ [28]	2012	30/30	T: 18–50, 35 C: 18–50, 35	T: 23/7 C: 22/8	KS + TLV	TLV	48/NR	0	HBeAg loss; HBV-DNA; ALT norm; ALT; TER

Yang and Zeng [29]	2013	43/42	T: 18–60, 30 C: 18–62, 31	T: 25/18 C: 25/17	KS + TLV	TLV	48/NR	0	HBeAg sero; HBV-DNA

^*∗*^The different authors with the same name in English.

KS: Kushenin, 0.6 g/d; LAM: lamivudine, 0.1 g/d; ETV: entecavir, 0.5 mg/d; ADV: adefovir dipivoxil, 10 mg/d; TLV: telbivudine, 600 mg/d.

HBeAg loss: hepatitis B e antigen loss; HBsAg loss: hepatitis B s antigen loss; HBeAg sero: hepatitis B e antigen seroconversion; HBV-DNA: undetectable HBV-DNA levels; ALT norm: normalization of serum alanine aminotransferase levels; AST norm: normalization of serum aspartate transaminase levels; ALT: serum ALT level; AST: serum AST level; TBIL: serum TBIL level; TER: total efficacy rate (serum level of ALT decreases at least 25% and serum level of AST decreases at least 50%).
